# Retrospective Assessment of Metabolic Syndrome and Cardiovascular Disease Risk Following Monthly and Three-Month Long-Acting Paliperidone Palmitate Treatment in Schizophrenia

**DOI:** 10.1192/j.eurpsy.2024.460

**Published:** 2024-08-27

**Authors:** S. N. Ispir, E. Yıldız, H. R. Demirel, H. Söylemez, M. Aydın

**Affiliations:** Psychiatry, Selcuk University Faculty Of Medicine, Konya, Türkiye

## Abstract

**Introduction:**

Patients with schizophrenia exhibit a higher prevalence of metabolic syndrome and cardiovascular diseases compared to the general population, resulting in increased mortality rates. The extent of this risk may vary based on the specific treatment employed.

**Objectives:**

This study aims to compare the risk assessments of metabolic syndrome and cardiovascular diseases in schizophrenia patients who transitioned from monthly long-acting paliperidone palmitate (PP1M) treatment to three-month long-acting paliperidone palmitate (PP3M) treatment during both treatment periods.

**Methods:**

The research was conducted at the Psychiatry Clinic and Psychotic Disorders Outpatient Clinic of Selcuk University Faculty of Medicine. Eligible participants included patients under PP3M treatment for a minimum of 6 months and undergoing continuous monitoring in the psychotic disorders outpatient clinic. Sociodemographic and clinical data, scales, laboratory values, and measurements taken both before and during the use of PP3M and PP1M were retrieved from file records, encompassing assessments, analyses, and examinations conducted in accordance with the “Psychotic Disorders - Treatment Monitoring Protocol.” Ethical approval was obtained from the Selcuk University Ethics Committee.

**Results:**

Among the 31 patients transitioning from PP1M to PP3M treatment, 15 (48.4%) were female. The mean age of the patients was 44.4±14.4 years. No statistically significant differences were observed in the mean values of clinical evaluation and side effect assessment scales, body mass index (BMI), waist-to-hip ratio, systolic blood pressure, glucose levels, cholesterol levels, prolactin levels, and thyroid-stimulating hormone (TSH) measurements between the pre- and post-treatment phases (p>0.05). However, a significant difference was identified in the mean Qrisk3 values, a cardiovascular risk index, in two distinct measurements (10-year risk score: PP1M 3.7±4.2 and PP3M 4.6±4.8, p=0.003).

**Conclusions:**

Our study, designed to investigate the impact of the monthly and three-month long-acting formulations of the same antipsychotic drug on patients’ clinical status, side effects, and general health parameters, found that PP3M treatment did not significantly differ from PP1M treatment in terms of Qrisk3 values. Despite the lack of statistical significance between the parameters used in Qrisk3 calculation, the observed significant difference in Qrisk3 values is attributed to variations in age. In order to promote the widespread adoption of long-acting treatments in schizophrenia management, clinicians should engage in comprehensive comparative studies assessing both efficacy and side effects.

**Disclosure of Interest:**

None Declared

